# Concomitant fractures in older patients with proximal femoral fractures: rib fractures are associated with increased complications and mortality for consideration in european geriatric medicine

**DOI:** 10.1007/s41999-026-01603-x

**Published:** 2026-09-07

**Authors:** Clemens Roitzsch, Franziska Beyer, Klaus-Dieter Schaser, Marcel Mäder, Lorenz C. Hofbauer, Babette Engler, Roman Riedel, Ivonne Panchyrz, Anne Postler

**Affiliations:** 1 University Center of Orthopedic, Trauma and Plastic Surgery, University Hospital Carl Gustav Carus, TU Dresden, Fetscherst. 74, 01307 Dresden, Germany; 2 University Center for Healthy Aging & Division of Endocrinology, Diabetes, and Bone Diseases, University Hospital Carl Gustav Carus Dresden, TU Dresden, Fetscherst. 74, 01307 Dresden, Germany; 3 Center for Evidence-Based Healthcare, University Hospital Carl Gustav Carus and Faculty of Medicine Carl Gustav Carus, TU, Fetscherst. 74, 01307 Dresden, Germany

**Keywords:** Proximal femur fracture, Concomitant fracture, Geriatric, Mortality, Thrombosis, Rib fracture

## Abstract

**Purpose:**

Proximal femoral fractures (PFF) are a global health burden in aging populations. Evidence on the prevalence and clinical relevance of concomitant fractures in this population remains limited. This study investigated the impact of concomitant fractures on mortality, complications, transfusion requirements, and postoperative mobility in patients with geriatric PFF.

**Methods:**

This single-center cohort study included patients ≥ 65 years with a positive geriatric screening, who underwent surgery for a PFF after low-energy trauma between January 2019 and December 2023. Patients with concomitant fractures were compared with those with isolated PFF. Outcomes included mortality, perioperative parameters, complications, and mobility at discharge. Uni- and multivariable regression models were applied adjusting for age, sex, comorbidities, body mass index (BMI), and fracture type.

**Results:**

A total of 1553 patients were analyzed; 164 (10.5%) sustained concomitant fractures (*n* = 180), most commonly of the upper limb (57%), pelvis (16%), and ribs (13%). Concomitant fractures were ipsilateral in 89% and surgically treated in 29%. Compared with isolated PFF, these patients had higher rates of in-house venous thromboembolism (VTE) (5.5% vs. 0.9%, *p* < 0.001), required 0.4 more units of blood transfusion (95% confidence interval (CI) 0.114–0.744), and showed impaired mobilization at discharge (odds ratio (OR) 0.5, 95%CI 0.328–0.654). No difference in overall in-house or 90-day mortality was observed. However, concomitant rib fractures markedly increased the risk of non-surgical complications (OR 2.8, 95%CI 1.175–6.804), VTE (OR 14.8, 95%CI 4.078–53.492), blood transfusion by 1.1 units (95%CI 0.323–1.925), and 90-day mortality (OR 6.4, 95%CI 2.675–15.469).

**Conclusion:**

Concomitant fractures occur in over 10% of geriatric PFF patients and worsen clinical outcomes, particularly VTE risk, transfusion needs, and mobility. Rib fractures are a high-risk subgroup requiring specific clinical attention.

## Introduction

Proximal femur fractures (PFF) represent a major global health burden, particularly among older patients [1]. Due to demographic changes and an aging population, the incidence of fragility fractures in older patients, particularly PFF, is expected to constantly increase [2]. In 2019, over 155,000 PFF were reported in Germany alone, resulting in an increase of 23% compared to 2009 [3]. The estimated annual costs are 2–4 billion euro in Germany and 17 billion US dollar in the USA [4, 5]. Apart from the high treatment costs, PFF are associated with impaired quality of life, permanent disability, and high mortality rates, averaging 20% at 1 year [6–8].

Falls leading to PFF may also cause additional injuries, particularly in frail and multimorbid older patients. These concomitant fractures increase care complexity and may adversely affect outcomes, but existing evidence is limited. Studies investigating concomitant injuries in geriatric patients with PFF are scarce and mostly limited to retrospective single-center cohorts focusing on concomitant upper limb fractures [9–13]. An analysis from the German Registry for Geriatric Trauma in 2021 involving almost 25,000 patients aged 70 years or older with surgically treated proximal femur or peri-/interprosthetic fractures, revealed a prevalence of concomitant injuries of 8.2% (including fractures, dislocations and traumatic brain injuries (TBI)). Patients with concomitant injuries had a longer length of hospital stay, a prolonged time to surgery, and a higher in-house mortality [14]. Numerous studies have analyzed concomitant upper limb fractures, whereas, apart from this study, only few have addressed the overall impact of concomitant fractures of other regions [15–18].

This study aimed to investigate whether patients with low energy-related PFF and concomitant fractures differ regarding their individual characteristics, in-hospital and 90-day mortality, length of stay, complications, and postoperative mobility from those in patients with isolated PFF. Moreover, a subgroup analysis was performed to compare the impact of specific types of concomitant fractures on the outcome.

## Material and methods

### Patient selection and selected parameters

This single-center cohort study included all patients aged 65 years and older with a positive geriatric screening and a PFF after low-energy trauma who underwent surgical treatment at our academic level 1 trauma center in Germany between January 2019 and December 2023. Ethics approval for this study was granted by the Ethics Committee of the Medical Faculty Carl Gustav Carus, TU Dresden in 2021 (BO-EK 488102021).

All patients aged 65 years and older with a PFF after low-energy trauma were screened in the emergency department for geriatric status. Geriatric patients were identified based on predefined criteria. A positive screening required the presence of at least one of the following: age ≥ 85 years, an assigned care level, nursing home residency, or preexisting diagnosis of dementia [19]. The used Geriatrie-Check is a locally validated German screening tool for geriatric patients. In its German validation study, its screening performance was comparable to that of the internationally established and validated identification of seniors at risk (ISAR) screening tool [19]. Exclusion criteria were conservative treatment, isolated greater trochanteric fractures, periprosthetic or pathological fractures, revision surgery for previously treated fractures, polytrauma, and high-energy trauma.

For patients treated in 2019 and 2020, data collection was performed ambispectively. Patients were retrospectively identified from hospital records, and geriatric screening was reconstructed based on chart reviews. Patients or their relatives were subsequently contacted to actively ascertain 90-day survival.

Since January 2021, patient inclusion and data collection have been conducted prospectively.

In this analysis, particular attention was given to acute concomitant fractures sustained in the same accident that led to PFF and present at the time of admission. These were identified from clinical documentation and radiological reports. Concomitant fractures were categorized into six anatomical regions (subgroups): skull, upper limb, rib cage, spine, pelvis, and lower limb (excluding the index fracture). Both the presence and the number of affected regions were documented along with the side of the injury and need for surgical treatment. Besides, the incidence of TBI was recorded.

Collected data included demographic information (age, sex, BMI), comorbidities (ASA classification, medication), pre-fracture mobility, and fracture morphology.

### Therapy

Surgical treatment was performed by experienced orthopedic surgeons, with the type of the selected procedures based on individual fracture morphology and patient characteristics. Femoral neck fractures (AO31B) were treated with either total-hip arthroplasty (THA) or hemi-arthroplasty (HA), depending on age, pre-fracture activity level, comorbidities, and grade of preexisting osteoarthritis. Intertrochanteric (AO31A) and subtrochanteric fractures were treated using intramedullary fixation, i.e., cephalomedullary interlocking nailing systems (PFN-A, DePuy Synthes), with optional cement augmentation of the femoral neck blade or subtrochanteric cable cerclage as needed.

Anesthesia technique (general, spinal, regional, or a combination) was chosen based on patient comorbidities and anticoagulant use.

### Outcomes

In addition to time to surgery, hemoglobin concentrations 3 days postoperatively and required blood transfusion, we recorded postoperative mobility, mobilization, length of hospital stay, and in-hospital complications. Complications were recorded at discharge using a standardized form and included both surgical and non-surgical events (e.g., delirium, pneumonia, acute kidney injury, in-house venous thromboembolism (VTE)). In-house and 90-day mortality were documented through the hospital information system and follow-up by phone or mail with patients and relatives. Patients were divided into a concomitant fracture group and an isolated PFF group.

### Statistical analysis

For descriptive analyses, continuous variables were presented as median values with interquartile ranges and categorical data as frequencies and percentages. Statistical analysis included comparison between the groups of patients with concomitant fractures and patients with isolated PFF using the Mann–Whitney U test for continuous and ordinal variables and Chi-squared test for categorical data.

To assess the impact of concomitant fractures, binary logistic regression models were used to analyze dichotomous endpoints (e.g., the presence of certain complications or 90-day mortality). In addition, linear regression models were used for continuous endpoints (e.g., length of hospital stay) and ordinal regression models were used for ordinal endpoints. All models were adjusted for potential confounders such as gender, age, ASA score, BMI, and fracture type. The results were reported as odds ratio (OR) for logistic and ordinal regression models and as regression coefficient (ß) for linear regression models along with their 95% confidence intervals (CI). A p-value < 0.05 was considered statistically significant. Data were analyzed using SPSS (version 28.0 for Windows).

## Results

Overall, 1,893 patients aged 65 or older with PFF were screened during the study period. Of these, 1,555 fulfilled the predefined criteria for geriatric patients and low-energy trauma. Two patients were lost to follow-up, resulting in a final cohort of 1553 geriatric patients with PFF. Among them, 164 patients had at least one concomitant fracture (10.5%). Tables 1 and 2 provide patient characteristics and perioperative parameters for both groups. Patients with concomitant fractures were more often female (79.3%, *p* < 0.001) and had a median age of 85.2 years. Otherwise, patients with and without concomitant fracture were comparable in terms of age, comorbidities, BMI, pre-fracture mobility and fracture morphology.

**Table 1:** Patient characteristics with and without a concomitant fracture

		Overall		Isolated PFF *n* = 1389		Concomitant fracture *n *= 164		*P*-value
Median age, years		86.2	[81.7; 90.7]	86.3	[81.5; 90.7]	85.2	[80.0; 90.0]	0.165
Female, n		1030	66.3%	900	64.8%	130	79.3%	** < 0.001 †**
ASA-grade	252	16.2%	222	16.0%	30	18.3%	16.2%	0.448
	1301	83.8%	1167	84.0%	134	81.7%	83.8%	
BMI, kg/m^2^		23.7	[21.2; 26.6]	23.8	[21.3; 26.6]	23.4	[21.2; 26.7]	0.136
Fracture type								
Femoral neck fracture, n		693	44.6%	620	44.6%	73	44.5%	0.976
Intertrochanteric fracture, n		860	55.4%	769	55.4%	91	55.5%	
Anesthesia								
General, n		537	34.9%	468	34.0%	69	42.1%	0.054
Spinal, n		409	26.6%	372	27.0%	37	9,0%	
Regional, n		78	5.1%	75	5.5%	3	3.8%	
Combination, n		516	33.5%	461	33.5%	55	10.7%	
Antithrombotics, n		508	32.8%	466	33.6%	42	25.6%	**0.039 †**
Vitamin K antagonist, n		31	2.0%	29	2.1%	2	1.2%	0.450
DOAC, n		457	29.5%	419	30.2%	38	23.2%	0.062
Walking aids prior fracture								
None, n		598	44.8%	564	44.7%	34	46.6%	0.963
Forearm crutch, n		88	6.6%	84	6.7%	4	5.5%	
Rollator		545	40.8%	517	41.0%	28	38.4%	
Wheelchair		70	5.2%	66	5.2%	4	5.5%	
Bedbound		34	2.5%	31	2.5%	3	2.5%	
Mobility prior fracture								
>500m, n		191	14.3%	172	13.6%	19	26.0%	0.217
50–500m, n		345	25.8%	331	26.2%	14	19.2%	
Ward mobility, n		389	29.1%	370	29.3%	19	26.0%	
Room mobility, n		329	24.6%	314	24.9%	15	20.5%	
Bedbound/standing, n		81	6.1%	75	5.9%	6	8.2%	

Bold values indicate statistical signficance (*P* < 0.05)

Median [interquartile ranges], absolute (relative) frequencies, † chi-squared test

**Table 2:** Perioperative parameter for patients with and without a concomitant fracture

		Overall		Isolated PFF *n* = 1389		Concomitant fracture *n* = 164		*P*-value
Median erythrocyte concentrates total		0	[0; 2]	0	[0; 2]	1	[0; 2]	**0.003 ‡**
Medial Hb 3d postoperative		5.6	[5.0; 6.3]	5.7	[5; 6.3]	5.4	[4.7; 6.2]	**0.014 ‡**
Surgical complications, n		44	2.8%	40	2.9%	4	2.4%	0.748
Non-surgical complication, n		268	17.3%	236	17.0%	32	19.5%	0.419
VTE, n		22	1.4%	13	0.9%	9	5.5%	** < 0.001 †**
Median time to surgery, hrs		22.3	[15.0; 31.1]	22.1	[14.9; 30.8]	23.4	[16.5; 36.6]	0.054
Length of hospital stay, days		9	[7; 13]	9	[7; 12]	10	[8; 14]	**0.032 ‡**
In-house mortality, n		65	4.2%	57	4.1%	8	4.9%	0.640
90-day mortality, n		309	19.9%	273	19.7%	36	22.1%	0.470
Walking aids at discharge								
None, n		1	0.1%	1	0.1%	0	0.0%	0.286
Forearm crutch, n		167	13.2%	145	12.2%	22	31.0%	
Rollator, n		866	68.5%	837	70.2%	29	40.8%	
Wheelchair, n		102	8.1%	93	7.8%	9	12.7%	
Bedbound, n		128	10.1%	117	9.8%	11	15.5%	
Mobility at discharge								
Ward mobility, n		668	46.6%	622	47.8%	46	35.1%	** < 0.001 ‡**
Room mobility, n		383	26.7%	352	27.1%	31	23.7%	
Bedbound/standing, n		381	26.6%	327	25.1%	54	41.2%	

Bold values indicate statistical signficance (*P* < 0.05)

VTE in-house venous thromboembolism, median [interquartile ranges], absolute (relative) frequencies, † chi-squared test, ‡ Mann–Whitney U test

Table 3 gives an overview about the types of fracture. In total, 164 patients suffered 180 fractures with 13 having multiple concomitant injuries (≥  3 injuries in total). Most common were upper limb (*n* = 102, 57%), followed by pelvis (*n* = 30, 16%) and rib fractures (*n* = 23, 13%). Patients with concomitant upper limb fractures had significantly better preoperative mobility, compared to those with isolated PFF (*p* = 0.010). Concomitant fractures occurred on the same side as the index fracture in 89% and were treated operatively in 53 of 180 cases (29%). 22 of the 53 cases (41.5%) were managed during the same surgical session as the PFF. Median time to surgery in a separate operation was 5 days. 40 operations were performed because of concomitant upper limb fractures, with 19 and 12 operations for distal radius and proximal humerus fractures, respectively. Four patients with rib fractures and pneumohemothorax were treated with a chest tube, four pelvic fractures underwent surgery (three sacroiliac screws, one hip replacement with acetabular reconstruction for an acetabular fracture on the same side). Three surgeries were performed because of cervical spine injuries and one for a lumbar spine fracture. One orbital fracture was treated with an osteosynthesis. Additionally, 12 TBI were observed, of which one received an intracranial drainage. Surgical treatment of concomitant fractures had no influence on 90-day mortality (*p* = 0.271).

**Table 3:** Overview of concomitant injuries. In total, there were 180 fractures in 164 patients (10.5%)

Concomitant fracture	*n* = 180	
Upper limb	102	57%
Pelvis	30	16%
Rib	23	13%
Spine	16	9%
Skull	5	3%
Lower limb	4	2%

During hospital stay, a VTE occurred in 5.5% of patients with concomitant fractures, compared to only 0.9% in the isolated PFF group. Median time to surgery for PFF was 23.4 h for the concomitant fracture group and 22.1 h for the isolated PFF group with a median length of stay of 10 and 9 days, respectively. More than 40% of the patients with concomitant fractures could be mobilized only to a standing position at most (bedbound and standing combined), compared to 25% in the isolated PFF group. Nearly 50% of patients in the isolated PFF group were able to walk along the ward at discharge, whereas this applied to 35% of those with concomitant injuries.

Table 4 provides an overview of the non-surgical complications.

**Table 4:** Overview of non-surgical complications between the groups

	Isolated PFF	Concomitant fracture
Non-surgical complications	236	32
Electrolyte disturbances	14	3
Cardiac complications	29	3
Pulmonary complications	50	6
Renal complications	44	4
Delirium	50	3
Venous thromboembolism	13	9
Gastrointestinal bleeding	9	0
Other complications	56	7

*PFF* proximal femur fracture

In-house mortality was 4.1% in the isolated PFF group and 4.9% in the concomitant fracture group (*p* = 0.640). The corresponding 90-day mortality rates were 19.7 and 22.1%, respectively (*p* = 0.470).

The impact of concomitant fractures on different outcome parameters was tested using logistic, ordinal and linear regression models as appropriate, adjusted for sex, age, ASA-score, BMI and type of fracture (Table 5). After adjustment, no difference in time to surgery or length of stay was discovered. Concomitant fractures in general were associated with 0.2 mmol/l lower postoperative Hb concentration (95% confidence interval (CI) − 0.354 − 0.052), an average increase of 0.4 units of packed red blood cells (95%CI 0.114–0.744) as well as a higher risk of VTE (odds ratio (OR) 5.8, 95%CI 2.423–14.058) and a significantly impaired mobilization at discharge (OR 0.5 for higher mobilization levels, 95%CI 0.328–0.654). Concomitant rib fractures were associated with an additional transfusion requirement of 1.1 units (95%CI 0.323 – 1.925) as well as a higher risk for non-surgical complications during hospital stay (OR 2.8, 95%CI 1.175 – 6.804), VTE (OR 14.8, 95%CI 4.078 – 53.492) and 90-day mortality (OR 6.4, 95%CI 2.675 – 15.469).

**Table 5:** Impact of concomitant fractures on perioperative parameters

	OR/ß	95% Confidence interval	*P*-value
Time to surgery, hours*			
All concomitant fractures	−5.861	(− 16.150; 4.427)	0.264
Pelvis	7.713	(− 14.374; 29.800)	0.493
Spinal	5.442	(− 21.668; 32.552)	0.694
Hb 3d postoperative, mmol/l*			
All concomitant fractures	−0.208	(− 0.365; − 0.052)	**0.009**
Rib	−0.441	(− 0.756; 0.012)	0.057
Pelvis	−0.372	(− 0.782; − 0.100)	**0.011**
Spinal	−0.619	(− 1.136; − 0.103)	**0.019**
Erythrocyte concentrates, unit*			
All concomitant fractures	0.429	(0.114; 0.744)	**0.008**
Rib	1.124	(0.323; 1.925)	**0.006**
Pelvis	0.753	(0.042; 1.465)	**0.038**
Upper limb	0.156	(− 0.234; 0.546)	0.434
Non-surgical complications‡			
All concomitant fractures			0.419
Rib	2.828	(1.175; 6.804)	**0.020**
VTE‡			
All concomitant fractures	5.837	(2.423; 14.058)	** < 0.001**
Rib	14.770	(4.078; 53.492)	** < 0.001**
Pelvis	3.099	(0.573; 16.753)	0.189
Spinal	5.236	(0.623; 43.990)	0.127
Upper limb	2.065	(0.623; 6.842)	0.236
Length of stay, days*			
All concomitant fractures	0.086	(− 2.128; 2.300)	0.939
Rib	1.245	(− 7.721; 10.210)	0.785
Upper limb	−0.187	(− 2.825; 2.452)	0.890
Mobilization at discharge#			
All concomitant fractures	0.464	(0.328; 0.654)	** < 0.001**
Rib	0.420	(0.166; 1.065)	0.068
Pelvis	0.472	(0.207; 1.075)	0.074
Upper limb	0.529	(0.343; 0.815)	**0.004**
90-days mortality‡			
All concomitant fractures			0.470†
Rib	6.433	(2.675; 15.469)	** < 0.001**
Pelvis	1.683	(0.696; 4.071)	0.248
Upper limb	0.624	(0.332; 1.173)	0.143

Bold values indicate statistical signficance (*P* < 0.05)

*PFF* proximal femur fracture, *VTE* venous thromboembolism, *Hb* Hemoglobin Concentration

*linear regression model, #ordinal regression model, ‡logistic regression model, OR/ß odds ratio/regression coefficient as appropriate, †chi-squared test

## Discussion

This prospective single-center study including more than 1500 patients aged 65 years or older with a positive geriatric screening who underwent surgery for a low-energy proximal femur fracture demonstrated that concomitant fractures are common, occurring in more than 10% of the patients. While no association was found between concomitant fractures in general and 90-day mortality, surgical or non-surgical complications, concomitant rib fractures increased the patient’s risk of death. Moreover, concomitant fractures in general, as well as rib fractures were significantly associated with a higher incidence of VTE. Length of stay with a concomitant fracture was longer in univariable comparisons, but not after multivariable adjustment.

While much research has addressed PFF, studies analyzing concomitant fracture remain limited, often focused only on upper limb injuries and mostly retrospective. #We found upper limb injuries in 6.6%, which is comparable to the literature. Several retrospective studies reported similar findings, with concomitant upper limb fractures occurring in 4–7% of PFF patients and being associated with prolonged hospitalization, but with no consistent effect on mortality [13, 20–22]. However, most of these studies had a lower age threshold and none had a specific screening for geriatric status. A recent systematic review investigated the impact of concomitant upper limb fractures in PFF patients [23]. Concomitant upper limb fractures were recorded in 6.2% and associated with a longer length of stay and higher 30-day mortality, but no difference thereafter. The longer length of stay in most studies could be explained by a delayed and complicated mobilization in the presence of concomitant fractures. This may be due to additional pain associated with the concomitant fracture and increased challenges in mobilization, for example when the use of a walking aid is hindered by an upper limb fracture. This could also result in the discovered higher risk for VTE.

While the present study also showed a higher incidence for VTE and transfusion rates for patients with concomitant fracture, no association between in-house or 90-day mortality and upper limb fractures was found. Given the high baseline 90-day mortality in our cohort (19.9%), consistent with recent prospective findings [24] (19.7%), it is conceivable that this may have limited the detectability of any incremental risk associated with concomitant upper limb fractures. Length of stay was only significantly different in univariate analysis but not after adjusting for cofounders. The systematic review did not access in-house mortality, the two national database studies [21, 22] showed a higher in-house mortality in contrast to our findings. However, median age in those cohorts was notably lower than in ours (86 vs. 81 and 80 years, respectively) and unlike the mentioned studies, our cohort was defined by a positive geriatric screening rather than solely by an age-based inclusion criterion. Moreover, the considerably larger sample size of more than 200,000 patients might have rendered smaller differences between groups statistically significant. In our study, we observed higher, but not significant, rates of in-house mortality among patients with concomitant fractures (4.1 vs. 4.9%).

As shown in this study, mobilization for patients with concomitant fracture was significantly impaired with over 40% of patients only being able to mobilize to a standing position at most and 35% were mobile on the ward. In line with these results, a study from the German Geriatric Trauma Registry comparing pre- and postoperative (7 days) walking ability showed significantly worse results for patients with concomitant fractures vs. isolated PFF [14].

Generally, only few studies investigated more than one specific concomitant injury in PFF patients. Smaller single-center analyses reported mostly lower rates of concomitant fractures (4–5%) without differences in complications or mortality [14–17]. However, two of the studies used an age cutoff of 60 years, one study did not apply any age cutoff, and none of the studies employed an additional geriatric screening. The previously mentioned analysis from the German Geriatric Trauma Registry analyzed over 22,000 patients 70 years and older with PFF or periprosthetic fracture and found concomitant injuries in 8.2% [14]. They discovered a prolonged time to surgery and extended length of stay in patients with concomitant injuries, which is in-line with our study. Contrary to our results, concomitant fractures of spine, pelvis, TBI, and concomitant fractures generally were associated with a higher in-house mortality, but only TBI had a persistent impact on mortality after 120 days. However, the present study included all intracranial bleedings, with almost 70% conservative treatment, while the German Geriatric Trauma Registry only includes severe TBI. This may also be attributed to the comparably generous indication for performance of cranial CT scans in our institution, which could have led to the detection of also smaller and asymptomatic hemorrhages with milder clinical presentation, that otherwise would have been unrecognized.

Additionally, in former studies, concomitant rib fractures were not analyzed separately. In our investigation, rib fractures were significantly linked to a higher 90-day mortality. To the best of our knowledge, no further literature specifically addresses concomitant rib fractures in PFF patients. Particularly in our vulnerable geriatric patient cohort, rib fractures may lead to reduced respiratory excursion, pain, and an increased risk of complications such as pneumonia. In this context, appropriate management—including intensive respiratory physiotherapy—is of crucial importance to reduce rib fracture-/pain-induced susceptibility to pneumonia and prevent adverse outcomes.

A recent prospective UK study reported a lower incidence of concomitant injuries (4.1%), likely due to the exclusion of vertebral and rib fractures. Their results otherwise aligned with ours, showing prolonged hospital stay but no difference in mortality [17].

Interestingly, concomitant upper limb fractures occurred significantly more frequently among patients with better pre-fracture mobility, which is in line with previous research [25]. One possible explanation is that patients with better pre-fracture mobility may also have superior orientation and protective reflexes during a fall. Consequently, the upper limb fracture may result from an attempt to mitigate the impact by means of arms and hands during stumbling/fall before hitting the ground [26, 27]. Supporting this hypothesis, nearly 90% of concomitant fractures occurred on the ipsilateral side.

Some limitations need to be noted. Although this was a study with a large sample size, it was conducted at a single level I trauma center. Therefore, our findings may not be generalizable to all hospitals or healthcare systems in other countries. During the first 2 years, the study followed an ambispective design with retrospective patient identification, which may have introduced a potential risk of information bias compared with the subsequent fully prospective data collection. The number of patients with concomitant rib fractures in our cohort was relatively small (*n* = 23). While the observed associations were consistent and clinically plausible, they should be interpreted with some caution and ideally confirmed in larger, multicenter studies. Of note, we did not formally screen for osteoporosis using dual-energy X-ray absorptiometry (DXA). However, given that PFF from low-energy trauma in a cohort of this age typically reflects an osteoporotic fracture and the majority of associated fractures were also major osteoporotic fractures, we assume that most fractures were indeed osteoporotic. Preoperative mobility status was available for only 81% of patients; therefore, a change (delta) in mobility between pre- and postoperative period could not be reported. Besides, we did not differentiate between postoperative mobilization to standing and bedbound status. Furthermore, we were unable to obtain information on readmissions to other hospitals, and only in-hospital complications were recorded. The Geriatrie-Check used to define our study population is a locally validated German screening tool, which may limit the generalizability of our findings to populations assessed using other geriatric screening instruments. However, its performance was shown to be comparable to the internationally established ISAR screening tool [19]. In addition, most previous studies investigated unselected or differently selected proximal femoral fracture populations without additional geriatric screening. These differences in patient selection limit direct comparisons between studies and should be considered when interpreting similarities or differences in reported outcomes.

In addition, preexisting comorbidities were not assessed in detail and only captured using the ASA score. As comorbidities may independently influence posttraumatic mortality—e.g., by increasing susceptibility to VTE or by predisposing to falls and impaired protective reflexes—the limited information on comorbidities represents an important limitation of this study.

## Conclusion

Concomitant fractures in patients with proximal femoral fractures are common, occurring in more than 10% of cases. While concomitant fractures in general increase the risk of VTE, blood transfusion and impair postoperative mobility, they do not affect 90-day mortality. To the best of our knowledge, this is the first study to demonstrate an association between concomitant rib fractures and an increased risk of non-surgical complications, VTE, and 90-day mortality. Physicians should be aware of these findings and carefully assess for concomitant fractures in order to ensure timely diagnosis and appropriate management, including preventive measures to decrease non-surgical complications and proactively improve the course of disease in these vulnerable patients.

## Data Availability

The data that support the findings of this study are not openly available due to reasons of sensitivity and are available from the corresponding author upon reasonable request. Data are located in controlled access data storage at the University Center for Orthopedics, Trauma and Plastic Surgery, University Hospital Dresden.
